# Immunohistochemical Detection of MYC-driven Diffuse Large B-Cell Lymphomas

**DOI:** 10.1371/journal.pone.0033813

**Published:** 2012-04-12

**Authors:** Michael J. Kluk, Bjoern Chapuy, Papiya Sinha, Alyssa Roy, Paola Dal Cin, Donna S. Neuberg, Stefano Monti, Geraldine S. Pinkus, Margaret A. Shipp, Scott J. Rodig

**Affiliations:** 1 Department of Pathology, Brigham and Women's Hospital, Boston, Massachusetts, United States of America; 2 Department of Medical Oncology, Dana-Farber Cancer Institute, Boston, Massachusetts, United States of America; 3 Department of Biostatistics, Dana-Farber Cancer Institute, Boston, Massachusetts, United States of America; 4 Division of Computational Biomedicine, Boston University, Boston, Massachusetts, United States of America; University of Nebraska – Lincoln, United States of America

## Abstract

Diffuse large B cell lymphoma (DLBCL) is a clinically and genetically heterogeneous disease. A small subset of DLBCLs has translocations involving the *MYC* locus and an additional group has a molecular signature resembling Burkitt lymphoma (mBL). Presently, identification of such cases by morphology is unreliable and relies on cytogenetic or complex molecular methods such as gene transcriptional profiling. Herein, we describe an immunohistochemical (IHC) method for identifying DLBCLs with increased MYC protein expression. We tested 77 cases of DLBCL and identified 15 cases with high MYC protein expression (nuclear staining in >50% of tumor cells). All *MYC* translocation positive cases had increased MYC protein expression by this IHC assay. In addition, gene set enrichment analysis (GSEA) of the DLBCL transcriptional profiles revealed that tumors with increased MYC protein expression (regardless of underlying *MYC* translocation status) had coordinate upregulation of MYC target genes, providing molecular confirmation of the IHC results. We then generated a molecular classifier derived from the MYC IHC results in our cases and employed it to successfully classify mBLs from two previously reported independent case series, providing additional confirmation that the MYC IHC results identify clinically important subsets of DLBCLs. Lastly, we found that DLBCLs with high MYC protein expression had inferior overall survival when treated with R-CHOP. In conclusion, the IHC method described herein can be used to readily identify the biologically and clinically distinct cases of MYC-driven DLBCL, which represent a clinically significant subset of DLBCL cases due to their inferior overall survival.

## Introduction

The transcription factor and cell cycle regulator MYC (c-MYC) is a well-recognized oncoprotein in B-cell lymphoma. Nearly all Burkitt lymphomas (BLs) exhibit elevated MYC protein expression due to transcriptional deregulation following a balanced translocation involving *MYC* and, most commonly, the immunoglobulin heavy chain locus (*IgH*) [1]. In contrast, only 10% of diffuse large B-cell lymphomas (DLBCLs) harbor a *MYC* translocation [2]–[4]. However, additional DLBCLs that may not harbor a *MYC* translocation exhibit features of “molecular Burkitt lymphoma (mBL)” by gene expression profiling (GEP) [3], [5]. Although patients with DLBCL are typically treated with rituximab-containing CHOP-like chemotherapy regimens, the therapy for BL includes alternating combinations of more intensive multi-agent chemotherapy [6]–[8]. It is now recognized that the presence of a *MYC* rearrangement is an independent predictor of poor outcome in DLBCL patients who are treated with standard, DLBCL-directed therapy [2]–[4], [9]. Thus, the sub-classification of DLBCL based on *MYC* status has become critical for selecting patients who are candidates for more intensive, BL-type regimens. Presently, routine identification of cases of DLBCL with *MYC* abnormalities is not possible by morphology and, therefore, is dependent upon cytogenetic or complex molecular methods [3], [5], [10]. Herein, we report a standardized immunohistochemical (IHC) approach to assess MYC protein expression in formalin-fixed paraffin-embedded tissue (FFPE) which readily identifies DLBCLs with high nuclear MYC protein expression. Biologically, primary DLBCL cases with increased MYC protein expression exhibit coordinate upregulation of MYC target genes and have a poorer outcome following R-CHOP treatment. In addition, a molecular classifier derived from the gene transcriptional profiles of the MYC-driven DLBCLs identified by the immunohistochemical assay largely captures tumors classified as mBLs in previously reported series [3], [5].

## Methods

### Case selection

56 primary (*de novo*) DLBCLs from 2004–2009 with matched frozen and FFPE tissue as well as 21 secondary (recurrent or transformed) DLBCLs with FFPE tissue were identified through the Department of Pathology, Brigham and Women's Hospital and reviewed to confirm presence of diagnostic tissue and final diagnosis [11]. Clinical history, treatment and survival data were obtained from chart reviews with IRB approval. Cases for survival analysis had documentation of an R-CHOP based treatment regimen.

### Immunohistochemical Analysis

**Figure 1:**
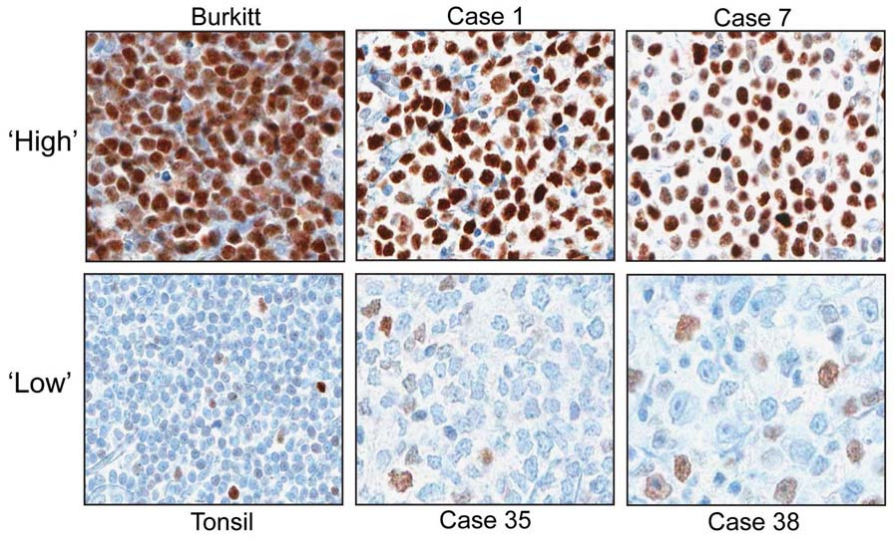
Immunohistochemical detection of MYC in representative Primary DLBCLs. Photomicrographs of select tumors and reactive tissue stained for MYC (positive staining = brown nuclei). Positive control (Burkitt lymphoma with a confirmed *MYC* translocation) revealed uniform, intense staining in >90% of tumor cells (Burkitt). In contrast, reactive lymphoid tissue revealed variable staining in only 10% of normal lymphocyte nuclei (Tonsil). Representative images from DLBCL cases and associated percent MYC+ tumor nuclei: Case 1, 90% MYC+; Case 7, 70% MYC+; and Cases 35 and 38, 30% MYC+. MYC staining was exclusively nuclear in all cases under the described staining conditions.

**Figure 2:**
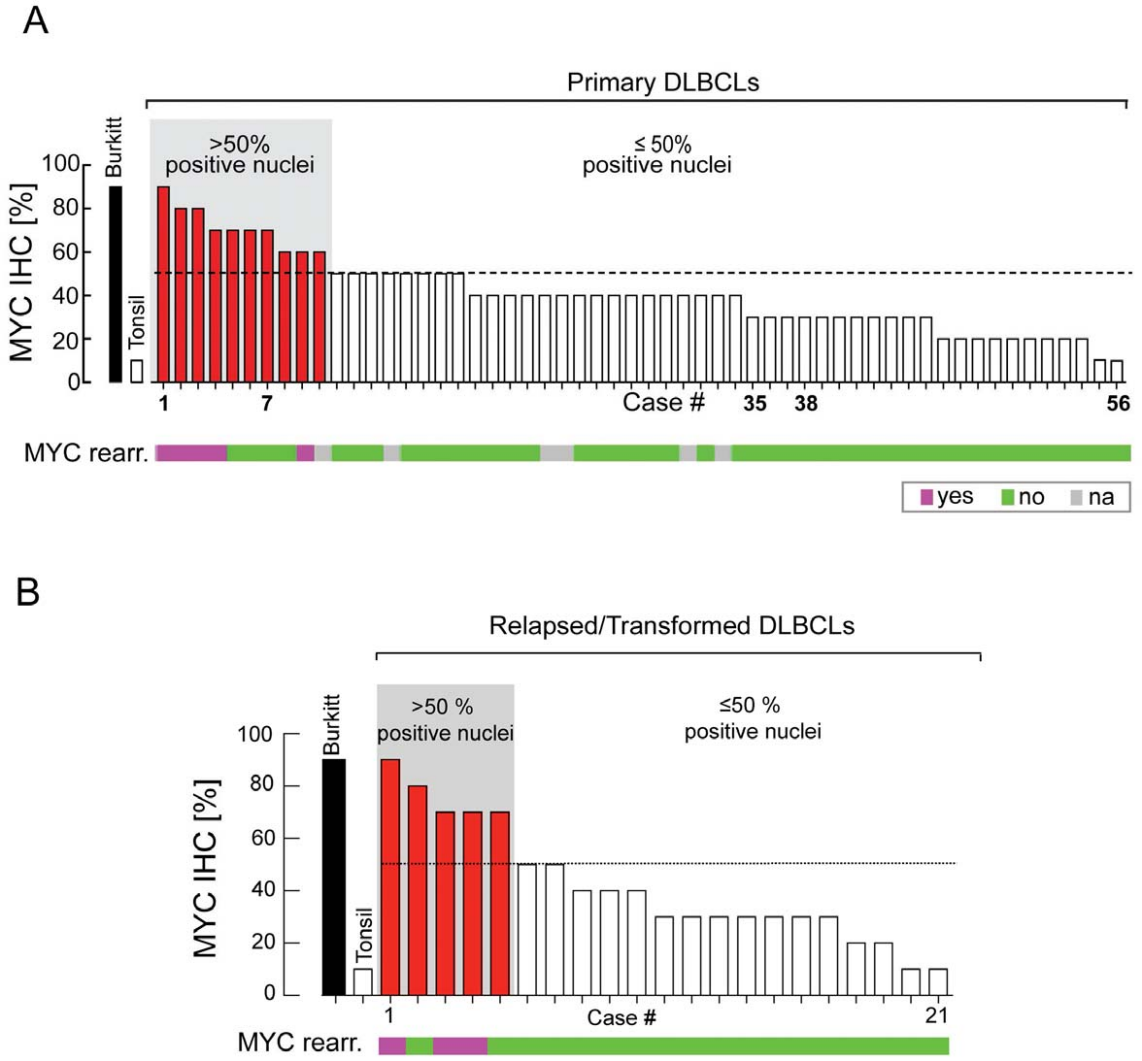
Comparison of MYC protein expression and *MYC* translocation status in cases of Primary *(de novo)* DLBCL; (**A**). Comparison of percent MYC positive tumor nuclei for Burkitt lymphoma, reactive tonsil and 56 primary DLBCLs with their corresponding *MYC* translocation status (bar graph; pink = *MYC* translocation, green = no *MYC* translocation, gray = not determined). Tumors with an IHC score of >50% are indicated in red at left. Tumors are arranged by IHC-determined percentage of MYC-positive nuclei. **Comparison of MYC protein expression and** ***MYC*** **translocation in cases of Secondary DLBCL;** (**B**). Comparison of percent MYC positive tumor nuclei for Burkitt lymphoma, reactive tonsil and 21 cases of recurrent or transformed DLBCLs with their corresponding *MYC* translocation status (bar graph; pink = *MYC* translocation, green = no *MYC* translocation). Tumors with an IHC score of >50% are indicated in red at left. Tumors are arranged by IHC-determined percentage of MYC-positive nuclei.

4 µm thick FFPE full tissue sections were stained for MYC (rabbit monoclonal anti-human MYC antibody; catalog #1472-1, Epitomics, Inc., Burlingame, CA, USA) [12] in the Specialized Histopathology Laboratory and the Anatomic Pathology Immunohistochemistry Laboratory (Department of Pathology, Brigham and Women's Hospital) on Ventana Benchmark XTs (Ventana Medical Systems, Tucson, AZ, USA) using extended antigen retrieval (CC1 buffer), anti-MYC antibody (final concentration 0.56 µg/ml) and signal amplification (mouse anti-rabbit reagent followed by rabbit anti-mouse reagent, Figure 1). The percentage of positive tumor nuclei was manually scored from 0 to 100% in 10% intervals and was also assessed with an Aperio ScanScope (Aperio Technologies Inc., Vista, CA), ImageScope software, and an optimized algorithm for nuclear staining (Nuclear V.9, Aperio, Inc.) [13]. Independent scoring by two hematopathologists showed very high concordance for final MYC classification (*kappa* statistic = 0.941). The scoring results from the pathologist (MJK) are represented in Figure 2. The Ki67 score was taken from the diagnostic pathology reports for the majority of cases. For those cases in which Ki67 was not performed at the time of diagnosis, the stain was performed as part of this study and interpreted by a pathologist (SJR) blinded to the genetics of the tumor.

### Genetic Analysis

*MYC* translocations were characterized according to standard and published protocols of karyotypic analysis and by fluorescent in situ hybridization (FISH) with a Vysis LSI MYC dual color, break apart rearrangement probe (catalog # 05J91-001, Abbott Molecular, Abbott Park, IL, USA) [14].

### Bioinformatic Analysis

Gene expression profiling of primary DLBCLs and GSEA analysis for MYC and 20 MYC target genes [5] were performed according to standard protocols as described (See Supplementary Methods S1) [14]–[16]. A molecular classifier derived from differential gene expression between the MYC IHC-High and MYC IHC-Low DLBCLs was generated according to previously published protocols [14](See Supplementary Methods S1).

## Results

IHC analysis of 56 primary DLBCL cases revealed a spectrum of total tumor cell nuclei (ranging from 10% to 90%) that stained positive for MYC by manual scoring (Figures 1 & 2A). Digital image analysis (Aperio ScanScope) revealed concordant findings with manual scoring (Figure S1). Cases in which the majority (>50%) of tumor nuclei stained positive for MYC (10 cases, Figures 1 & 2A) exhibited moderate to strong staining intensity, while cases in which ≤50% of tumor nuclei were MYC positive had dim to moderate staining intensity (46 cases, Figures 1 & 2A). All of the cases of primary DLBCL with a *MYC* rearrangement (9%; a frequency consistent with prior reports) [2] showed MYC staining in >50% of tumor nuclei (range of 60–90%, manual scoring) (Figure 2A). An additional 4 cases of primary DLBCL without a *MYC* translocation had increased MYC expression by IHC (Figure 2A). Likewise, when we assessed MYC staining in an independent cohort of 21 cases of secondary DLBCL, we found that all *MYC* translocation positive cases had >50% MYC positive tumor nuclei and that an additional two cases of secondary DLBCL without a *MYC* translocation also had increased MYC expression (Figure 2B).

**Figure 3:**
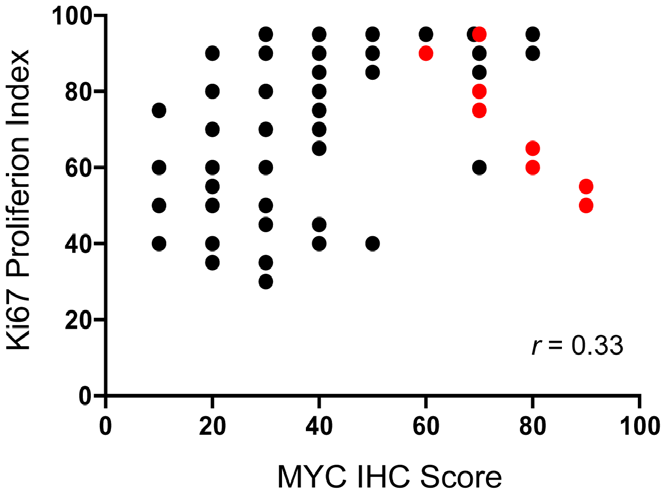
Comparison of MYC IHC score and Ki67 proliferation index for all cases analyzed. Cases with a *MYC*-translocation are colored in red and cases without a *MYC*-translocation are colored in black. The two biomarkers demonstrate a weak but positive correlation (Spearman *r* = 0.33, p = 0.003).

A subset of cases (n = 24) was re-stained an additional two times over a span of six months, once on the same staining instrument and once on a separate staining instrument in a distinct laboratory. Quantification of MYC positive tumor nuclei for each case by two independent pathologists revealed that the staining and the quantification procedure were highly reproducible (Figures S2; S3). A comparison of the MYC IHC scores with the Ki67 proliferation scores for the DLBCLs revealed only a weak correlation (Spearman *r* = 0.33, p = 0.003, Figure 3); indicating that the biomarkers are not redundant.

**Figure 4:**
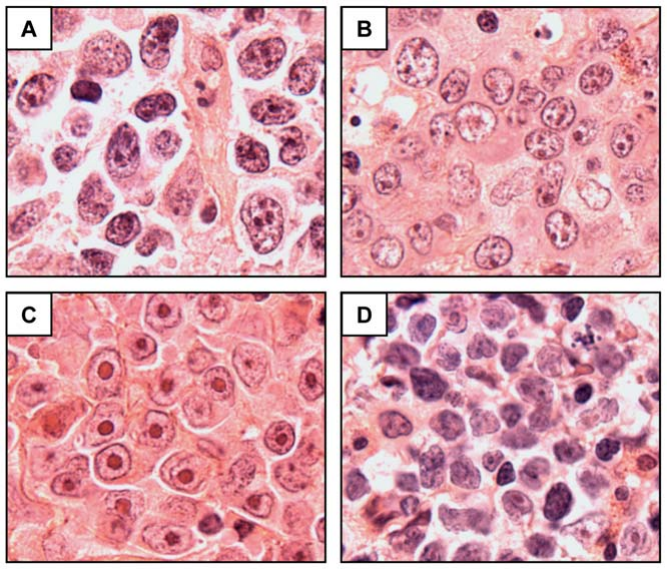
Morphological features of select MYC IHC-High DLBCLs. Hematoxylin and Eosin stained sections (all 1000× original magnification) of (A) Relapse case #2, DLBCL NOS; (B). Relapse case #4, DLBCL NOS; (C) Primary case #1, DLBCL, immunoblastic variant; (D) Primary case #3, DLBCL, NOS, with some morphological features that might suggest B-cell lymphoma, unclassifiable, with features intermediate between DLBCL and BL (see text for details).

We therefore defined two categories of DLBCLs for further analysis (i.e., those with >50% MYC-positive nuclei vs. those with ≤50% MYC-positive nuclei). This cut-point identified all cases with an underlying *MYC* translocation, in two independent cohorts. Tumors that scored as MYC IHC-High (>50% MYC-positive nuclei) showed a spectrum of immunophenotypic and morphological features (Figure 4, Table 1). Upon additional review, no cases matched criteria for BL and only 2 of 15 cases had some morphologic characteristics that might suggest as “B-cell lymphoma, unclassifiable, with features intermediate between DLBCL and BL” (Int. DLBCL/BL) by the 2008 WHO criteria (Figure 4D; Table 1). Although the 2 cases showed intermediate to large sized tumor cells with fine nuclear chromatin, the cases lacked a “starry sky” appearance at low magnification, the tumor nuclei showed marked pleomorphism, and the tumors exhibited immunophenotypes that are not consistent with BL (i.e., BCL2+) or Int. DLBCL/BL (i.e. Ki67<90%) and, therefore, are best classified as DLBCL, NOS.

**Table 1:** Pathological Features of MYC IHC-High DLBCL.

Case Number		MYC IHC (%)	*MYC* Trans-location?	IHC- Positive	IHC- Negative	Ki67 (%)	Morphological Diagnosis
1° DLBCL	2° DLBCL						
1		90	Yes	CD20, CD10, BCL6, BCL2		50	DLBCL-Immunoblastic
2		80	Yes	CD20, BCL6, BCL2	CD10	65	DLBCL-Immunoblastic
3		80	Yes	CD20, CD10, BCL6, BCL2		60	DLBCL, NOS
4		70	Yes	CD20, BCL6, BCL2	CD10	80	DLBCL, NOS
5		70	No	CD20, BCL2	CD10, BCL6	90	DLBCL-Immunoblastic
6		70	No	CD20, CD10, BCL6, BCL2		95	DLBCL, NOS
7		70	No	CD20, BCL6	BCL2, CD10	85	DLBCL, NOS
8		60	No	CD20, CD10, BCL6, BCL2		95	DLBCL, Anaplastic
9		60	Yes	CD20, BCL2	CD10	90	DLBCL, NOS
10		60	N/A	CD20, BCL6, BCL2	CD10	N/A	DLBCL, NOS
	1	90	Yes	CD20, CD10, BCL2	CD5	55	DLBCL, NOS
	2	80	No	CD20	CD10	90	DLBCL, NOS
	3	70	Yes	CD20, CD10, BCL6, BCL2		75	DLBCL, NOS
	4	70	No	CD20, CD10, BCL2		60	DLBCL, NOS
	5	70	Yes	CD20, BCL6, BCL2	CD10	95	DLBCL, NOS

**Figure 5:**
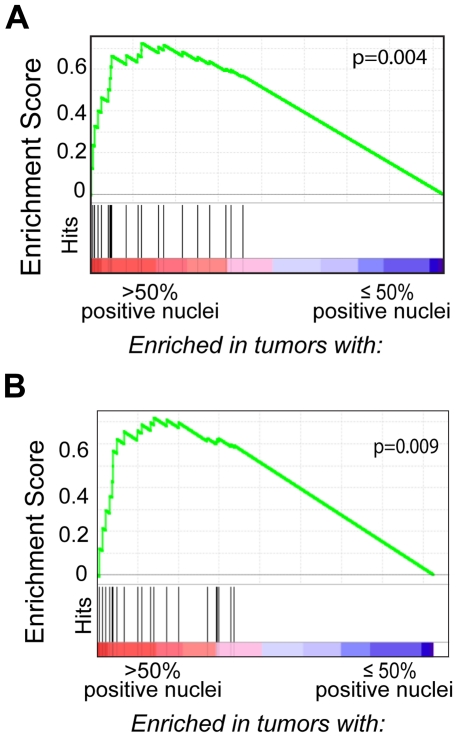
GSEA of MYC target genes in tumors characterized for MYC protein expression; (**A**). Gene Set Enrichment Analysis (GSEA) of all primary DLBCL cases reveals coordinate upregulation of MYC and MYC target genes (black vertical lines) in tumors with >50% MYC positive tumor nuclei (including *MYC* translocation-positive and –negative cases) (See Supplementary Methods S1 for additional details). **Gene Set Enrichment Analysis (GSEA) of MYC-target genes in** ***MYC*** **translocation–negative Primary DLBCL tumors;** (**B**) *MYC* translocation-negative primary DLBCL cases with >50% MYC positive tumor nuclei have coordinate upregulation of MYC and MYC target genes (black vertical lines).

Gene set enrichment analysis (GSEA) revealed that primary DLBCL cases with >50% MYC-positive tumor nuclei exhibited coordinate upregulation of MYC and MYC target genes [5] when compared to cases with ≤50% positive tumor nuclei (Figure 5A). Importantly, similar results were obtained when the GSEA was restricted to *MYC*-translocation negative cases with >50% MYC-positive tumor nuclei by IHC (Figure 5B), providing molecular confirmation that the increased MYC protein expression detected in these cases correlates with the upregulation of MYC and MYC target genes.

Two independent laboratories have reported that a subset of DLBCLs have a gene transcriptional profile resembling Burkitt lymphoma (“molecular Burkitt lymphoma”, mBL), and that these cases largely, but not completely, overlap with those tumors harboring a *MYC* translocation [3], [5]. We wished to determine whether DLBCLs with high levels of staining for MYC resemble those previously categorized as mBL by transcriptional profiling. Although we did not have access to the previously categorized tumor samples to directly test them by IHC, we developed an ensemble molecular classifier of MYC-driven DLBCL using the transcriptional profiles from a subset of our DLBCLs categorized as MYC IHC-High (i.e., >60% tumor cells positive) and MYC IHC-Low (i.e., <50% tumor cells positive) and applied this ensemble classifier to the previously published transcriptional profile datasets (Supplementary Methods S1). Our MYC IHC-derived molecular classifier correctly identified the vast majority of DLBCLs previously categorized as mBL from both series (17/19 cases, 89%) (Table 2, Tables S1 and S2) [3], [5]; thereby providing molecular evidence that MYC IHC can identify tumors with a transcriptional profile resembling mBL.

**Table 2:** Correlation of MYC IHC Gene Transcriptional Profile Classifier Result with mBL Signature.

MYC IHC-High/Low Transcriptional Profile	Dave^1^ DLBCL Signature		Hummel^2^ DLBCL Signature	
	mBL	Non-mBL	mBL	Non-mBL
**High**	8	2	9	7
**Low**	0	64	2	109
p-value^3^	<0.0001		<0.0001	

1) Series includes 74 cases of large B-cell lymphoma identified as having a Burkitt signature (mBL) or not (non-mBL) based on transcriptional profiles.

2) Series includes 127 cases of diffuse large B-cell lymphoma identified as having mBL or non-mBL based on transcriptional profiles. The MYC IHC High/Low gene expression classifier results for the cases that were identified as “Intermediate” by Hummel are included in Table S2.

3) Fisher's exact test.

**Figure 6:**
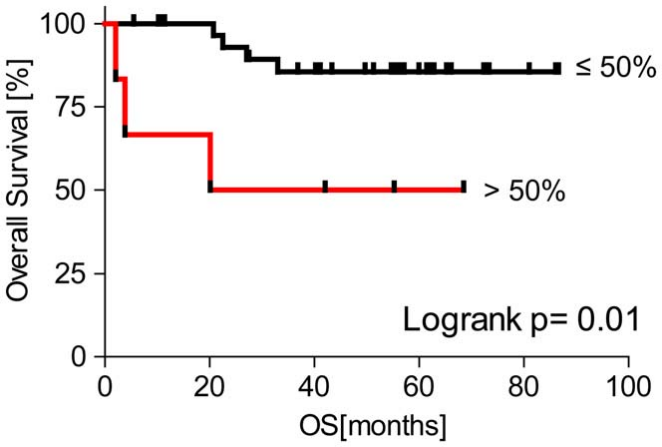
Outcome analysis according to MYC protein expression in Primary (*de novo*) DLBCL. Kaplan-Meier curve depicting the statistically significant (p = 0.01, log rank test) difference in the overall survival for the 38 primary DLBCL patients treated with R-CHOP. Tumors were grouped by MYC IHC score >50% (n = 6) or ≤50% (n = 32).

Lastly, in order to shed light on the clinical significance of DLBCL with high MYC protein detected by IHC, we compared the overall survival of patients with primary DLBCL whose tumors exceeded our threshold for MYC expression (>50%) with those below the threshold (≤50%). Among patients who were confirmed to have received R-CHOP-based therapy and had available long-term follow-up, those patients whose tumors had >50% MYC positivity had poorer overall survival compared to those with ≤50% (Figure 6). Three of the six tumors in the group exhibiting >50% MYC positivity harbored a *MYC* translocation and three did not. None of the tumors in the group with <50% MYC positivity harbored a *MYC* translocation (Table 3). Tumors in the cohort came from patients with a range of international prognostic index scores (IPI), however cases classified as MYC IHC-High were associated with a higher IPI than cases classified as MYC IHC-Low (p = 0.06, Kruskal-Wallis test, Table 3). The size of the patient cohort is not sufficient to determine whether high MYC expression is a poor prognostic marker independent of IPI (DSN, Department of Biostatistics, DFCI).

**Table 3:** Characteristics of R-CHOP treated Patient Cohort used for Survival Analysis.

Category	Subcategory	MYC High^1^^,^ 2	MYC Low^1^
Number of Patients		6	32
Gender – Female∶Male		4∶2	11∶21
Median Age [years] (range)		66 (50–73)	59 (27–81)
MYC Translocations		3	0
IPI	Low −0/1	1	15
	Low/Intermediate −2	1	4
	High/Intermediate −3	1	4
	High −4/5	3	4
	NA	0	5
Median Follow Up [months] (range)		31 (2–69)	55 (6–87)

1) MYC High cases show positive staining for MYC in >50% of tumor nuclei; MYC Low cases show positive staining for MYC in ≤50% tumor nuclei.

2) Cases classified as MYC IHC-High are associated with a higher IPI than cases classified as MYC IHC-Low (p = 0.06, Kruskal-Wallis test).

## Discussion

Diffuse large B cell lymphoma represents a clinically and genetically heterogeneous group of tumors. Various morphologic, immunohistochemical, cytogenetic and molecular subgroups have been identified [11]. One such important subgroup includes cases with a *MYC* translocation that occurs in approximately 10% of all cases and is associated with a poor prognosis [2]. Another clinically significant subgroup harbor a molecular signature resembling Burkitt lymphoma, and these cases are often referred to as molecular Burkitt lymphoma (mBL). In some reports, this subgroup has also been associated a poor prognosis [3], [5]. Currently, cases of DLBCL with a *MYC* translocation or mBL signature cannot be readily identified by morphologic or immunohistochemical features. [10]

Herein, we have described a standardized immunohistochemical method for detecting MYC protein expression that can readily identify cases of DLBCL with a *MYC* translocation. More specifically, we have found that cases of both primary and secondary DLBCL with a *MYC* translocation reproducibly have >50% of tumor nuclei which are strongly positive for MYC protein. Gene Set Enrichment Analysis (GSEA) of the primary DLBCLs with >50% MYC positive tumor nuclei correlates with upregulated MYC and MYC target genes, providing confirmation that the detection of a high MYC protein expression by IHC is associated with an activated MYC transcriptional profile.

A novel observation from this study is the broad range of MYC protein expression in DLBCL. We observed MYC IHC scores ranged from 10–90%, however for practical purposes, grouping tumors as MYC IHC-High versus MYC IHC-Low was desirable. We established a cut-off value for classifying tumors as the lowest MYC IHC score that captures all cases with a confirmed *MYC* translocation (>50% tumor nuclei positive for MYC). This decision was further justified by data indicating that this cut-point identified a group of patients with inferior clinical outcome when treated with R-CHOP (Figure 6). We also observe a statistically significant difference in clinical outcome when we raise the cut-point for classifying tumors as MYC IHC-High to >60% positive tumor nuclei (p = 0.001, Log-rank test). However when lower cut-points are used (i.e. MYC IHC >40%), we do not observe a statistically significant difference in outcome (DSN, Dept. Biostatistics, DFCI). It should be noted that our cohort of patients with outcome data is small (38 patients), and that these results will need to be validated in additional patient cohorts and across multiple institutions.

Unexpectedly, we found that the Ki67 proliferation index of the tumors showed only a weak correlation with the MYC IHC score (Spearman *r* = 0.33, p = 0.003, Figure 3, Table 1). This result suggests that Ki67 is not a good surrogate biomarker for MYC IHC, and that a high Ki67 proliferation index and a *MYC*-translocation are not as closely associated in DLBCL as they are in BL. This result is in agreement with some prior reports showing that Ki67 proliferation index is not a good predictor of *MYC*-translocation status in DLBCL. [9] [17]

An additional novel finding from this study is the identification of DLBCLs with high MYC protein in the absence of a *MYC*-translocation. Gene set enrichment analysis (GSEA) confirmed increased MYC transcriptional activity in MYC IHC-High DLBCLs lacking a *MYC*-translocation (Figure 5B), and thus, there must be alternative mechanisms for MYC deregulation. We found that one primary MYC IHC-High DLBCL case (Case #8) had copy number gain in *MYC* (6 copies), however the remaining cases had only 2 copies of *MYC*. Alternative cellular alterations resulting in increased MYC expression have been described for tumors other than DLBCL, and these include transcriptional and post-transcriptional deregulation [18], [19]. Determining the mechanisms that are responsible for MYC deregulation in MYC IHC-High DLBCLs lacking a *MYC*-translocation will be a focus of future efforts.

There has been much interest in tumors conforming to the pathological diagnosis of DLBCL but harboring a gene transcriptional profile resembling BL (“molecular Burkitt lymphoma”, mBL). A subset of mBL cases lacks a *MYC*-translocation and is not identified by routine pathologic or cytogenetic techniques. We did not have access to the primary tissue samples from the original case series describing mBL to test directly by IHC. However, we used the transcriptional profiles from a subset of our cases to derive an ensemble molecular classifier that we applied to the published transcriptional profiles of DLBCLs analyzed by Dave et al., and Hummel et al. It is important to note that in constructing our ensemble classifier, we used the transcriptional profiles from our set of DLBCLs with unequivocally high (>60%) and low (<50%) levels of MYC staining. Our classifier correctly identified the majority of DLBCLs previously classified as “mBL” (17/19 cases, 89%), and correctly identified the majority of DLBCLs previously classified as “Non-mBL” (173/182 cases, 95%). The results are highly significant (p<0.0001, Table 2), and indicate that MYC IHC can identify a set of tumors with transcriptional profiles resembling those previously categorized as mBL.

Nevertheless, our ensemble classifier did not capture all cases previously categorized as “mBL” or “Non-mBL” (Table 2). Two cases categorized as mBL in the Hummel series but categorized as “MYC IHC-Low Gene Transcriptional Profile” by our classifier were negative for a *MYC* translocation. Of the 7 cases classified as “Non-mBL” cases by the Hummel classification but classified as “MYC IHC-High Gene Transcriptional Profile” by our classifier, 2 cases had a *MYC*-translocation. Furthermore, one of the two previously reported series of cases (Hummel et al.) also included an “Intermediate mBL/non-mBL” category of DLBCL. When we applied our classifier to this category of tumors (Table S2), a subset of cases (10/38, 26%) sorted as having a “MYC IHC-High gene transcriptional profile”, and a subset of cases (28/38, 74%) sorted as having a “MYC IHC-Low gene transcriptional profile”. Direct staining of the primary tissues from these case series will be necessary to determine the true sensitivity and specificity of MYC IHC in identifying DLBCLs with a profile of mBL and to establish whether MYC IHC is robust enough to capture DLBCLs that classify as “Intermediate mBL/non-mBL”.

The WHO Classification of Tumours of Haematopoietic and Lymphoid Tissues (4th edition) includes the diagnostic category “B-cell lymphoma, unclassifiable, with features intermediate between diffuse large B-cell lymphoma and Burkitt lymphoma” (Int. DLBCL/BL) to encompass a heterogeneous group of tumors that do not meet the specific criteria of DLBCL or BL. [11] As defined, this category includes cases that morphologically resemble BL but exhibit atypical immunophenotypic or genetic profile, and cases with morphologic features overlapping BL and DBLCL but exhibit a phenotypic or genetic profile compatible with BL. At this time, this category does not include cases of morphologically typical DLBCL and have a *MYC*- rearrangement.

Each of the 77 cases examined in this study were originally diagnosed as “Diffuse large B-cell lymphoma, NOS” by the WHO Classification scheme. Re-examination of hematoxylin and eosin stained sections from our 15 cases scored as MYC IHC-High revealed a spectrum of morphological patterns consistent with typical DLBCL, NOS, for most cases. However we also observed cases with features consistent with the immunoblastic and anaplastic variants of DLBCL (Table 1, Figure 4). Only two cases showed intermediate to large cell size and fine nuclear chromatin that could raise the possibility of classifying the tumors as “B-cell lymphoma, unclassifiable, with features intermediate between DLBCL and BL” by the 2008 WHO criteria. However these cases lacked a “starry sky” appearance at low magnification, showed marked nuclear pleomorphism at high magnification (Figure 4D), did not demonstrate a high Ki67 proliferation index (60% and 75%, respectively) and had an abnormal immunophenotype (BCL2+) and, therefore we felt that that the classification as DLBCL, NOS, was appropriate. Overall, we did not find a unifying set of morphological or phenotypic features that would allow pathologists to identify cases of DLBCL with high MYC activity with certainty.

Despite the morphologic appearance of DLBCL, NOS, we find that patients with MYC IHC-High DLBCL do poorly when treated with R-CHOP. This result is consistent with results from others who have reported that patients with DLBCLs harboring a *MYC*-rearrangement exhibit inferior clinical outcome compared to those without a *MYC*-rearrangement when treated with standard chemotherapy. [2], [3], [9] Therefore, we believe that the WHO diagnostic category of Int. DLBCL/BL as currently defined may not fully capture cases of MYC-driven DLBCL. A more clinically relevant diagnostic category within the WHO might be “MYC-driven diffuse large B-cell lymphoma”. Adoption of such a category will require further discussion among expert pathologists and a careful retrospective analysis of the literature.

IHC is utilized for diagnostic purposes by a large proportion of pathology laboratories in the United States on a daily basis. However the technique and interpretation of the results are recognized as difficult to standardize. [20] One of the goals of this study is to provide a reproducible method for detecting and quantifying MYC staining in FFPE biopsy samples that can serve as a template for other pathology laboratories that may wish to implement the test.

To ensure precision in the automated staining protocol, we stained identical samples of tonsil, Burkitt lymphoma, and 22 DLBCL cases including the primary DLBCL case #9 (DLBCL with *MYC*-translocation near the cut-point for MYC IHC-High) three times. For two staining runs, the same staining instrument was used. For the third staining run, a separate staining instrument (also a Ventana Benchmark XT) located in a separate laboratory was employed. Microscopic examination of the slides revealed that the staining was reproducible, even when the staining runs were spaced months apart (Figure S2). Moreover, the final classification of each DLBCL tested did not change across staining runs. To establish the precision in quantifying the stained tumor samples, all cases were scored independently by two hematopathologists and by computer based image analysis algorithm (Figures S1; S3). We found very high inter-observer agreement between pathologists (*kappa* statistic = 0.941) and between the pathologists and the image analysis algorithm for the final tumor classification. Thus our data suggest that the results of this assay can be reproducible over time.

In implementing MYC IHC in any new laboratory, we suggest strict fidelity to the validated staining protocol described here and through the consistent inclusion of appropriate positive and negative control tissues whenever the assay is performed. Specifically, one should use the specific antibody clone (Y69, Epitomics Inc.) validated in this manuscript since other antibodies may perform differently. The antibody should be used at the final concentration stated (0.56 microg/mL) using the standard immunohistochemical staining platform and detection kit (Ventana Benchmark XT) as per our validation protocol for formalin fixed paraffin embedded tissues. Since the antibody concentration as supplied by the company varies from lot to lot it is important to use the same final concentration of antibody rather than the same dilution or titer. The provider of the antibody (Epitomics, Inc.) maintains the antibody concentration data for each lot and provides it upon request. Other automated staining platforms (i.e. Leica Bond, DAKO autostainer) are also likely to be amenable for detecting MYC in FFPE tissues, however the optimal final concentration of anti-MYC antibody may differ on these staining platforms and separate validations will need to be performed. [12]

Furthermore, we strongly suggest that positive and negative control tissues should be included in each staining run. These calibrators ensure the general technical success of the assay and serve as a guide for interpreting the stained samples of interest. For each staining run we always include reactive tonsil and Burkitt lymphoma with a confirmed *MYC* translocation to ensure that the antibody and staining platform performs as expected. We would further suggest, as a control tissue, inclusion of a known DLBCL with a *MYC* translocation at or near the cut-point for classification as MYC IHC-High. This will help establish the threshold for classification as MYC IHC-High across staining runs- especially when prospectively testing cases. In establishing the precision of our assay over multiple staining runs, we included the primary DLBCL case #9 (a DLBCL with *MYC*-translocation and 60% of the tumor nuclei positive for MYC staining, Figure 2A) as a control. Despite the consistency of MYC staining in our hands, we will continue to include the appropriate positive, negative, and threshold control cases, preferably the exact same cases to ensure the consistency of the results over time and recommend others do so as well. As with any new IHC procedure implemented within a CLIA-approved laboratory, each institution will need to complete an initial internal validation on a set of genetically defined DLBCLs (i.e. assessed for a *MYC* translocation by FISH) to determine the exact testing conditions required for their institution's tissue fixation and processing protocols.

We are implementing MYC IHC into our daily practice of diagnostic surgical pathology but, at least initially, as part of a prospective study to validate the findings from the current retrospective analysis. Our diagnostic pathology department, like a large proportion of pathology departments in the United States, is equipped with automated immunostainers. IHC is performed daily and on all cases of lymphoma. Therefore, implementation of MYC IHC is straightforward in our daily practice. MYC IHC will be included in the standard panel of immunohistochemical stains performed on each case of suspected DLBCL and the MYC IHC score determined manually by a diagnostic surgical pathologist. All cases of DLBCL will also undergo FISH analysis to assess for a *MYC*-translocation.

If a prospective analysis confirms the reliability of the test in general and the cut-point for positive staining tumor nuclei described here (>50% tumor nuclei positive for MYC), MYC IHC will be implemented as a screening test for deciding which cases of DLBCL we will further analyze by FISH in daily practice. With increasing use and familiarity with MYC IHC, it is our hope to reliably and prospectively identify patients with DLBCL and high MYC protein who may benefit from more aggressive chemotherapy, irrespective of their *MYC*-translocation status.

It is worth noting that although we used an image analysis algorithm to independently confirm the number of positive staining tumor nuclei obtained by manual quantification, we will use manual quantification in daily practice. Although computer-assisted image analysis is conceptually appealing as a means to quantify MYC staining in daily practice, in the current form the technique suffers limitations that are likely to limit general use. Specifically, computer-based analyses are unable to distinguish nuclei of tumor cells from those of admixed, reactive lymphocytes, endothelial cells, and other non-neoplastic cells. As a result, the algorithms can under-call the number of positive-staining tumor nuclei (Figure S1). This problem is especially relevant to tumors such as T-cell rich/histiocyte rich DLBCL. Moreover, despite the increasing use of slide scanners, there is no standard diagnostic pathology test which currently uses image analysis *in lieu* of manual quantification by an expert pathologist, even when the quantification is clinically important (i.e. enumerating CD34+ cells on bone marrow biopsies as a marker of blasts). Thus, manual quantification will remain our primary means to enumerate positive-staining tumor nuclei.

In conclusion, we describe a robust and broadly applicable IHC method for identification of increased MYC protein in FFPE from cases of DLBCL. We show that cases of primary DLBCL with >50% MYC-positive tumor nuclei have increased MYC activity as assessed by GSEA and have an inferior overall survival following R-CHOP treatment. Importantly, we show that this IHC test has the advantage of identifying DLBCLs with deregulated MYC expression which lack a *MYC* translocation and that a molecular classifier derived from the gene expression profile of our cases can capture tumors defined as mBL by other groups. Although these findings will require confirmation in additional, independent tumor cohorts, our results indicate the feasibility and utility of IHC staining for MYC that can be implemented as part of the standard diagnostic evaluation of DLBCL.

## Supporting Information

Figure S1 **Comparison of manual and automated analysis of MYC expression by IHC in primary DLBCL cases;** (**A**). Comparison of MYC IHC percent positive tumor nuclei for each primary DLBCL determined manually by a pathologist (grey bars) or by an image analysis algorithm using Aperio ImageScope software (black bars). The threshold for >50% and ≤50% staining is indicated (horizontal line). The shaded area separates cases with >50% positive tumor nuclei from cases with ≤50% positive tumor nuclei. **Comparison of manual and automated analysis of MYC expression by IHC in secondary DLBCL cases;** (**B**). Comparison of MYC IHC percent positive tumor nuclei for each secondary DLBCL determined manually by a pathologist (grey bars) or by an image analysis algorithm using Aperio ImageScope software (black bars). The threshold for >50% and ≤50% staining is indicated (horizontal line). The shaded area separates cases with >50% positive tumor nuclei from cases with ≤50% positive tumor nuclei.
(TIF)

Figure S2 **Reproducibility of MYC IHC staining on an automated platform.** Representative images of the indicated, identical sets of cases stained 6 months apart. All photomicrographs are 1000× original magnification.
(TIF)

Figure S3 **Reproducibility of MYC IHC quantification.** Comparison of the average MYC IHC percent positive tumor nuclei for each indicated case of DLBCL stained three times (twice on one automated staining machine; once on a separate automated staining machine in a distinct laboratory) and determined manually by two pathologists (grey and black bars, respectively). The standard deviation from the mean for the 3 tests is indicated.
(TIF)

Table S1 **Sub-classification of the Dave et al., DLBCL series by pathology review, global transcriptional profile, and the MYC IHC-High/Low transcriptional profile classifier.**
(XLSX)

Table S2 **Sub-classification of the Hummel et al., DLBCL series by pathology review, global transcriptional profile, and the MYC IHC-High/Low transcriptional profile classifier.**
(XLS)

Methods S1 **Supporting information.**
(DOC)
