# Supplementary figures and images for: Immunohistochemical Detection of MYC-driven Diffuse Large B-Cell Lymphomas
Source: PLoS One. 2012 Apr 12;7(4):e33813. doi: 10.1371/journal.pone.0033813 (PMC3325231; doi:10.1371/journal.pone.0033813)

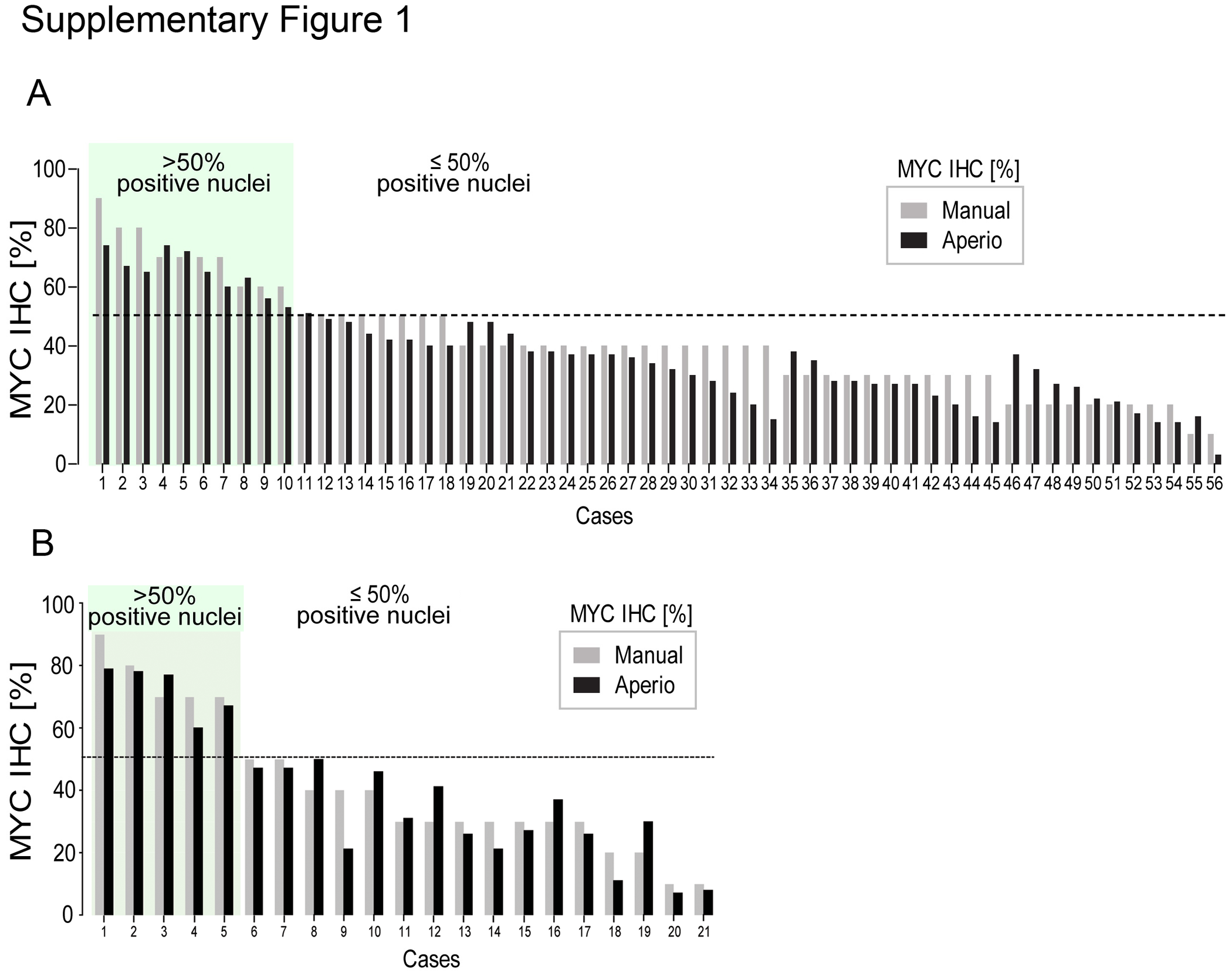

Supplement: Figure S1 — Comparison of manual and automated analysis of MYC expression by IHC in primary DLBCL cases; (A). Comparison of MYC IHC percent positive tumor nuclei for each primary DLBCL determined manually by a pathologist (grey bars) or by an image analysis algorithm using Aperio ImageScope software (black bars). The threshold for >50% and ≤50% staining is indicated (horizontal line). The shaded area separates cases with >50% positive tumor nuclei from cases with ≤50% positive tumor nuclei. Comparison of manual and automated analysis of MYC expression by IHC in secondary DLBCL cases; (B). Comparison of MYC IHC percent positive tumor nuclei for each secondary DLBCL determined manually by a pathologist (grey bars) or by an image analysis algorithm using Aperio ImageScope software (black bars). The threshold for >50% and ≤50% staining is indicated (horizontal line). The shaded area separates cases with >50% positive tumor nuclei from cases with ≤50% positive tumor nuclei. (TIF) [file pone.0033813.s001.tif]

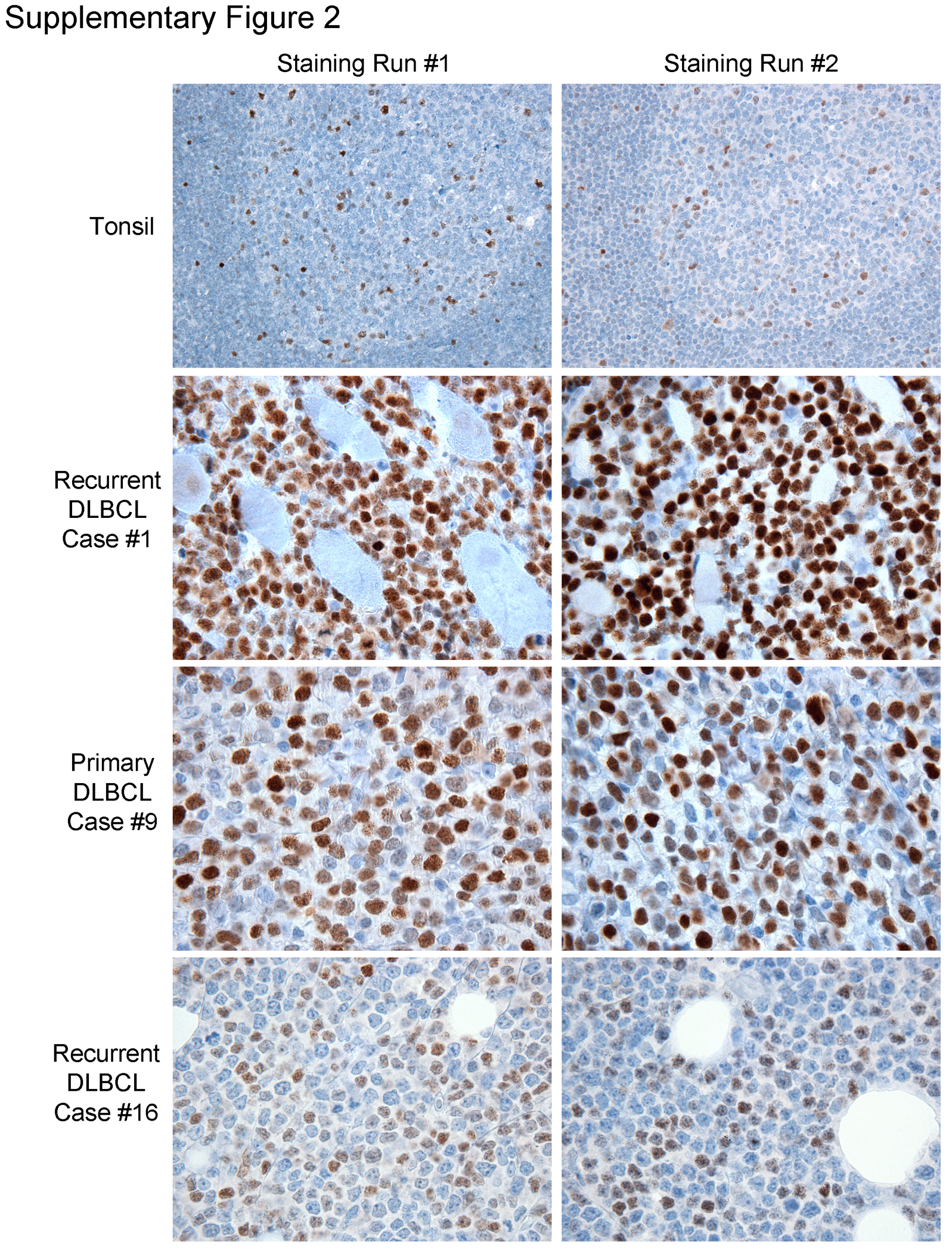

Supplement: Figure S2 — Reproducibility of MYC IHC staining on an automated platform. Representative images of the indicated, identical sets of cases stained 6 months apart. All photomicrographs are 1000× original magnification. (TIF) [file pone.0033813.s002.tif]

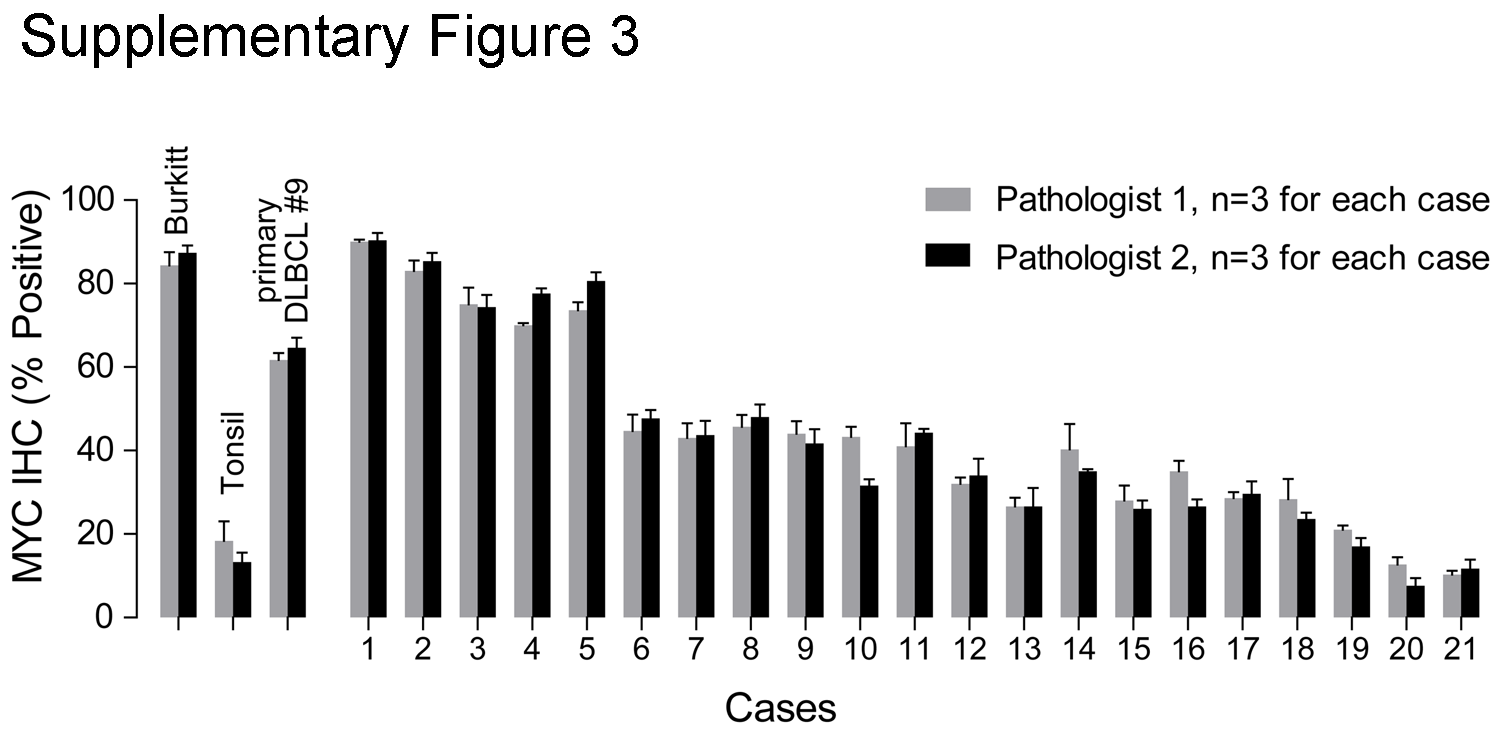

Supplement: Figure S3 — Reproducibility of MYC IHC quantification. Comparison of the average MYC IHC percent positive tumor nuclei for each indicated case of DLBCL stained three times (twice on one automated staining machine; once on a separate automated staining machine in a distinct laboratory) and determined manually by two pathologists (grey and black bars, respectively). The standard deviation from the mean for the 3 tests is indicated. (TIF) [file pone.0033813.s003.tif]
