# Supplementary material for: Immunohistochemical Detection of MYC-driven Diffuse Large B-Cell Lymphomas
Source: PLoS One. 2012 Apr 12;7(4):e33813. doi: 10.1371/journal.pone.0033813 (PMC3325231; doi:10.1371/journal.pone.0033813)
Supplement: Methods S1 — Supporting information. (DOC) [file pone.0033813.s006.doc]

**Immunohistochemical Detection of MYC-driven Diffuse Large B-cell Lymphomas**

# Supplementary Information

Michael J. Kluk, Bjoern Chapuy, Papiya Sinha, Alyssa Roy, Paola Dal Cin, Donna Neuberg, Stefano Monti, Geraldine S. Pinkus, Margaret A. Shipp, Scott J. Rodig

## Micro-array gene expression data processing

A set of well-curated primary DLBCL samples was profiled on Affymetrix U133_Plus2 chips. Probe-level data from the Affymetrix ‘.CEL’ files were summarized using the Robust Multi-array Average (RMA) procedure available through Bioconductor^1,2^, yielding a final data matrix of 53 samples by 54,675 probesets. Presence/absence calls were computed using the function mas5calls available in the Bioconductor package ‘Affy’.

## Gene-Set Enrichment Analysis

A set of 20 transcripts corresponding to a well-annotated list of 20 MYC targets^3^ was tested for enrichment against the MYC IHC phenotype by the GSEA method^4^. The complete series of 53 DLBCL primary samples was used for this analysis. Genes were sorted according to the value of the *t*-statistic computed against the MYC IHC “high *vs.* low” phenotype, with genes up-regulated in the “high” class at the left-end of the list, and genes up-regulated in the “low” class at the right-end of the list; the MYC targets were located within the sorted list, and their position was determined to be significantly skewed toward the “high” end of the sorted list by a weighted Kolmogorov-Smirnoff test^5^.

## Prediction of the IHC MYC phenotype on two independent datasets

13 distinct classifiers^5.6^ were trained on 44 of our 53 DLBCL samples, excluding cases with MYC IHC values between 50 and 60 (inclusive), and divided between MYC IHC 'high' and 'low' groups. The trained classifiers were then applied to two independent and publicly available datasets of 74 and 127 primary DLBCL samples arrayed on Affymetrix chips respectively^3,7^. Class membership (termed 'high' or 'low') was determined by a voting scheme whereby the vote of each classifier was weighted by the factor $w_{i}=e^{-5z_{i}}/W$, where *z_i_* is the *i*-thclassifier’s leave-one-one cross-validation (LOOCV) error rate on the training set, and$W=\sum_{i=1}^{13} w_{i}$. The number of predictive features (probe sets) used to build each of the 13 classifiers was determined by LOOCV in the training set. Supplementary Tables 1 and 2 report the ensemble class assignments and the corresponding weighted votes.

**Images and Statistics**

Stained slides were viewed and photographed with an Olympus BX41 microscope and Q-color5 digital camera (Olympus America, Inc., Center Valley, PA, USA) using Adobe Photoshop CS4 software (Adobe Systems, Inc., San Jose, CA, USA). Statistics, including Kaplan-Meier analysis, were performed with Graphpad Prism software (Graphpad Software, La Jolla, CA, USA).

**References**

1. Irizarry, R.A. et al. Exploration, normalization, and summaries of highdensity oligonucleotide array probe level data. Biostatistics. 2003;4:249-264.

2. Gentleman, R.C. et al. Bioconductor: open software development for computational biology and bioinformatics. Genome Biol. 2004;5:R80.

3. Dave, S.S. et al. Molecular Diagnosis of Burkitts Lymphoma. N Engl J Med. 2006;354:2431-2442.

4. Subramanian, A. et al. Gene set enrichment analysis: A knowledge-based approach for interpreting genome-wide expression profiles. Proc Nat Acad Sci. 2005;102:15545-15550.

5. Polo, J.M. et al. Transcriptional signature with differential expression of BCL6 target genes accurately identifies BCL6-dependent diffuse large B cell lymphomas. Proc Nat Acad Sci. 2007;104:3207-3212.

6. Chen, L. et al. SYK-dependent tonic B-cell receptor signaling is a rational treatment target in diffuse large B-cell lymphoma. Blood. 2008;111:2230-2237.

7. Hummel, M. et al. A Biologic Definition of Burkitts Lymphoma from Transcriptional and Genomic Profiling. N Engl J Med. 2006;354:2419-2430.
